# NUTRITIONAL STATUS AS A RISK FACTOR FOR DEPRESSION AMONG OLDER ADULTS RECEIVING COMMUNITY-BASED AGING SERVICES

**DOI:** 10.1093/geroni/igad104.3500

**Published:** 2023-12-21

**Authors:** Mary Goldsworthy, Timothy Boaz, Amber Gum, Lawrence Schonfeld

**Affiliations:** University of South Florida, Tampa, Florida, United States; University of South Florida, Tampa, Florida, United States; University of South Florida, Tampa, Florida, United States; University of South Florida, Tampa, Florida, United States

## Abstract

Nutritional status is a significant determinant of overall health and well-being among older adults and is linked to adverse mental health outcomes, such as depression. This study examined nutritional status as a risk factor for depression among 4,469 older adults receiving home- and community-based services from agencies overseen by a five-county Area Agency on Aging in west central Florida. It was hypothesized that older adults with a higher nutritional score (i.e., increased risk of malnourishment) would be more likely to have elevated depressive symptoms compared to those with lower nutritional risk. The cross-sectional study used secondary data from the AAA, including demographic and social variables, a 20-point nutritional risk score, and the Patient Health Questionnaire-9 (PHQ-9; not depressed = 0-9, depressed = >10) from clients in the AAA planning and service area. In logistic regression, the most significant predictor of depression was the client’s nutrition score (OR= 1.22, p < 0.0001). This OR suggests that, for every 1-point increase of the nutrition score, the odds of being depressed increases by 22%. The findings suggest that older adults receiving aging services with worse nutritional status are at increased risk of depression, consistent with other studies. The findings suggest that programs focused on improving nutritional status among older adults may reduce depressive symptoms may improve and may potentially improve long-term physical and mental health outcomes and decrease healthcare burden.

